# ASSOCIATION BETWEEN COGNITIVE FRAILTY AND THE RISK OF FALLS AMONG OLDER ADULTS: A SYSTEMATIC REVIEW

**DOI:** 10.1093/geroni/igad104.2275

**Published:** 2023-12-21

**Authors:** Shanshan Wang, Yueheng Yin, Isaac Sze Him Leung

**Affiliations:** The Hong Kong Polytechnic University, Hong Kong, Hong Kong; Nanjing Medical University, Nanjing, Jiangsu, China (People’s Republic); The Chinese University of Hong Kong, Hong Kong, Hong Kong

## Abstract

**Background:**

Falls lead to numerous negative health outcomes and jeopardize the physical function and quality of life in older adults. Cognitive impairment and physical frailty were found to be associated with the risk of falls, but there was no systematic review that estimated the association between cognitive frailty and the risk of falls.

**Methods:**

A systematic literature search of the cross-sectional, cohort, and case-control studies in Cochrane library, Scopus, CINAHL, EMBASE, and PsycINFO was conducted. Study quality was assessed by using the Joanna Briggs Institute (JBI) critical appraisal tool. A random effects meta-analysis was performed to estimate the odds ratio (OR) of the incidence of falls in older adults with cognitive frailty.

**Results:**

Seven studies were included. The overall quality of the studies was acceptable. The meta-analysis of cohort studies showed older adults aged 60 and above with cognitive frailty had a pooled OR of 1.45 (95%CI 1.30, 1.61) for at least one fall compared with those without cognitive frailty. The meta-analysis of cross-sectional studies showed that the odds of older adults with cognitive frailty experiencing at least one fall was 1.64 times (95%CI 1.51, 1.79) higher than those without cognitive frailty.

**Conclusion:**

Cognitive frailty is significantly associated with the risk of falls. Timely detection of cognitive frailty is essential for preventing falls in older adults

